# Antibody and antibody fragments site-specific conjugation using new Q-tag substrate of bacterial transglutaminase

**DOI:** 10.1038/s41420-024-01845-3

**Published:** 2024-02-15

**Authors:** Meddy El Alaoui, Eva Sivado, Anne-Catherine Jallas, Lamia Mebarki, Michael R. Dyson, Franck Perrez, Sandrine Valsesia-Wittmann, Said El Alaoui

**Affiliations:** 1 Covalab, 1B Rue Jacques Monod, 69500 Bron, France; 2 Centre Léon Bérard, INSERM 1296 Radiations : défense, Santé et environnement, 28 rue Laennec, 69008 Lyon, France; 3 IONTAS Ltd, Babraham Research Campus, Babraham, Cambridge, CB22 3AT UK; 4 Institut Curie, PSL Research University, CNRS UMR144 Paris, France

**Keywords:** Breast cancer, Biotechnology, Biochemistry

## Abstract

During the last few years Antibody-Drug Conjugates (ADCs) have become one of the most active and very promising therapeutic weapons. Lessons learned from the traditional chemical conjugations (via lysine or cysteine residues of the antibodies) and the clinical studies of the developed ADCs have recently paved the way to the improvement of the conjugation technologies. Use of site-specific conjugation is considered as the promising path for improving the design and development of homogeneous ADCs with controlled Drug-Antibody ratio (DAR). Moreover, some of these conjugations can be applied to antibody fragments such as Fab, scfv and VHH for which random and chemical conjugation showed significant limitations. In this study, we identified a novel small peptide substrate (Q-tag) with high affinity and specificity of bacterial transglutaminase which can be genetically fused to different formats of antibodies of interest for the development of enzymatic site-specific conjugation we named “CovIsolink” platform. We describe the synthesis of chemically defined drugs conjugation in which the site and stoichiometry of conjugation are controlled using a genetically encoded Q-tag peptide with specific amino acids which serves as a substrate of bacterial transglutaminase. This approach has enabled the generation of homogeneous conjugates with DAR 1,7 for full IgG and 0,8 drug ratio for Fab, scfv and VHH antibody fragments without the presence of significant amounts of unconjugated antibody and fragments. As a proof of concept, Q-tagged anti Her-2 (human IgG1 (Trastuzumab) and the corresponding fragments (Fab, scfv and VHH) were engineered and conjugated with different aminated-payloads. The corresponding Cov-ADCs were evaluated in series of in vitro and in vivo assays, demonstrating similar tumor cell killing potency as Trastuzumab emtansine (Kadcyla®) even with lower drug-to-antibody ratio (DAR).

## Introduction

Antibody-drug conjugates (ADCs) are the fastest growing class of anticancer agents. Adopting Paul Ehrlich’s “magic bullet” idea, they were designed to selectively deliver highly potent cytotoxic payloads to the malignant cells [1]. They combine the advantages of therapeutic monoclonal antibodies (mAbs) and the chemotherapeutic compounds. In this manner, the target specificity of mAbs enables the potent acceleration of tumor reaching drugs while leaving the normal cells largely unaffected, thus decreasing the off-target toxicity and increasing the therapeutic window [2]. There is considerable progress and success in this field, the number of ADCs entering to clinical trials are rapidly increasing. Nevertheless, so far, only 14 ADCs have been approved for oncologic indication by the US Food and Drug Administration (FDA) [3]. The majority of the immunoconjugates are produced by the conventional and non-specific chemical conjugations of surface exposed lysine (via activated esters) or interchain cysteine (maleimide chemistry) residues, as a result of heterogeneous mixtures [4]. Thus, each subpopulation exhibits distinct pharmacokinetic (PK) properties.

The potential extension of the therapeutic index and the in vivo performance is limited by the heterogeneity of ADCs [5]. Further attempts are ongoing in order that ADCs can deliver their full potential and improve the manufacturing challenges. Recently several site-specific conjugation technologies have been developed to control the drug-to antibody ratio (DAR), reduce the heterogeneity and batch-to batch variability. Based on the antibody scaffold we can distinguish two main strategies: (1) the selective conjugation is achieved by novel linker chemistry using the native mAb [6]. (2) Molecular engineering is required to introduce the coupling site into the mAb sequence such as mutation of cysteine residues [5, 7], glycoengineering [8] or insertion of unnatural amino acids [9] or peptide sequences for enzymatic approaches [10–14].

Recent studies have demonstrated microbial transglutaminase (mTG) can be an efficient tool for the production of site-specific homogeneous ADCs. mTG belongs to the group of enzymes that catalyze the post-translational modification of proteins by the formation of isopeptide bonds. This occurs either through protein crosslinking between the γ-carboxiamide group of glutamines and ε-amino groups of lysine residues or through incorporation of primary amines at selected peptide-bound glutamine residues [15–17]. The cross-linked products are highly resistant to chemical or proteolytic degradation. Since 1980s, m TG, a 38 kDa enzyme derived from Streptomices mobaraensis, was widely used, firstly, in food industry to improve the quality and shelf life of certain foods [18] then it was extended to other applications to cross-link polymers, small organic compounds and proteins due to its broad substrate specificity [19, 20].

The first use of mTG to generate ADCs was described by Strop et al. using genetically engineered peptide sequence (LLQG) [10]. Then other approaches were used to conjugate drugs after deglycosylation of the antibody or introducing a single amino acid mutation (N297Q) [12, 13]. However, it should be considered, that the absence of N-glycosylation can reduce the plasma-half life and alter the stability and aggregation rate of the ADCs.

In this study, we report the development of CovIsoLink™ (Covalently Isopeptide Crosslinking) technology for the generation of homogeneous ADCs using novel and advanced mTG-substrate sequences engineered into the heavy chain C-terminal of Trastuzumab antibody. While transglutaminases display broad amine donor substrate specificity, they are more selective towards the reactive glutamine donor residue [18, 21]. Herein we addressed to improve the mTG-mediated conjugation by the optimization of glutamine-containing peptide substrates. Our group has previously designed a synthetic peptide library to enhance their reactivity and specificity in comparison with the well-known smallest substrate peptide (ZQG) and its analog peptide LLQG [20, 22, 23]. The full length Trastuzumab and its corresponding fragments Fab, scfv and VHH, were conjugated with different payloads and evaluated in series of in vitro and in vivo models. Even with lower DAR, they showed similar potency than Kadcyla® used in the clinic.

## Results

### Generation of site-specific immunoconjugates

**Fig. 1:**
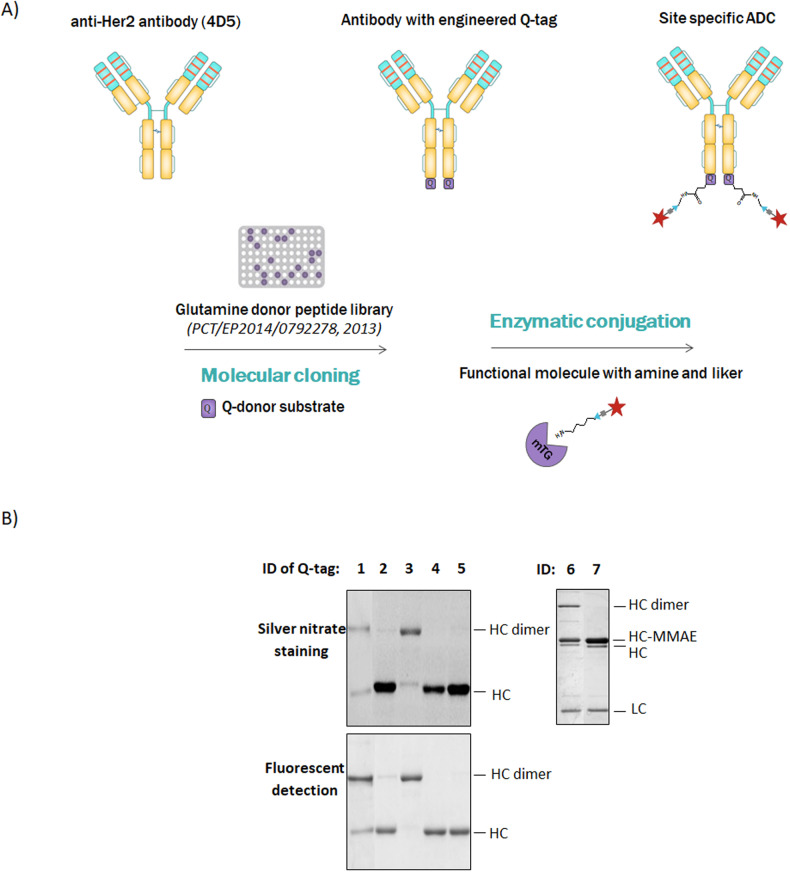
Microbial transglutaminase mediated site-specific conjugation of antibody drug conjugates. **A** Schematic representation of antibody with engineered glutamine tag (Q-Tag) and microbial transglutaminase mediated conjugation. **B** SDS-PAGE analysis of AlexaFluor488-cadaverine or MMAE conjugated engineered Trastuzumab-Qtag antibodies: 1-LLQG, 2-Tag2, 3-Tag3, 4-Tag4, 5-Tag5. ID 6: Trastuzumab-Qtag2-MMAE; ID 7: TrastuzumabK453del-Qtag2-MMAE. The reaction products were separated by 10% SDS-PAGE, then visualized by fluorescent detection and silver-nitrate staining. LC light chain, HC heavy chain, HC dimer heavy chain dimer.

Since transglutaminases are much selective toward the reactive glutamine than toward the lysine residue or primary amine, we aimed to improve the mTG technology by the optimization of the glutamine donor sequence (Q-tag). Previously we have created and screened (by transglutaminase colorimetric activity assay) [24] a synthetic glutamine donor peptide library [22]. Among 90 different peptides analyzed, four displayed higher reactivity (low apparent K_*M*_ values) compared to LLQG which has been identified as the best substrate of the enzyme [25] and used for the site-specific conjugation of engineered antibodies with Q-tag [10] (Fig. 1A). The 4 selected peptides were engineered into the C-terminal sequence coding for the heavy chains of the Trastuzumab targeting HER2/neu receptor. The conjugation efficacy was evaluated using AlexaFluor488-cadaverin as a payload, followed by SDS-PAGE analysis and fluorescent detection (Fig. 1B). Based on the intensity of the fluorescent signal, Tag2 (lane 2), Tag3 (lane3), Tag4 (lane 4) and Tag5 (lane 5) tagged antibodies showed higher signal intensity compared to LLQG (lane 1), suggesting better efficacy of coupling. For the further applications Q-tag_2_ (Tag2, lane 2) was selected.

SDS-PAGE analysis revealed that the product of high molecular weight corresponds to the dimerization of the heavy chains (Fig. 1B, lane 3 & 6). In silico analysis revealed the presence of lysine residues in the heavy chain C-terminal, known as amine donor substrate for mTG and can be responsible for interchains-crosslinking (10). Deletion of lysine was performed to generate Trastuzumab_K453del_-Qtag_2_ that was compared to wt (Fig. 1B, lane 6 and 7) leading to monomer ADCs only with increased conjugation yield 1.74 drugs per antibody vs 1,5 (Table 1). The acceptance of mTG was lower toward PEG_4_-SMCC-DM1 (no cleavable linker), resulted in an average DAR of 1.25 upon the current reaction conditions (Table 1). These Immunoconjugates were used for the further experiments.

**Table 1:** DAR evaluation by mass analysis of various trastuzumab constructs and payloads.

Antibody	Payload	Linker	DAR
*Trastuzumab-Qtag*_*2*_	Alexa 488-cadaverine		2
*Trastuzumab-Qtag*_*2*_	MMAE	PEG_4_-VC-PAB	1.5
*Trastuzumab*_*K453del*_*-Qtag*_*2*_	MMAE	PEG_4_-VC-PAB	1.74
*Trastuzumab-Qtag*_*2*_	DM1	PEG_4_-SMCC	1.25

The conjugation of structurally different amine containing payloads required unique optimization. The Immunoconjugates were analyzed by mass spectrometry to determine the drug attachment in the antibody (Supplementary Fig. 1). The coupling of AlexaFluor488-cadaverine resulted in the maximum yield in the expected DAR of 2. For different tubulin inhibitor drugs, we obtained comparable conjugation efficacy. 75% of heavy chains were converted with PEG_4_-VC-PAB-MMAE (cleavable linker), the mixture of DAR1 and DAR2 was detected without the presence of any unconjugated antibodies (Table 1).

**Fig. 2:**
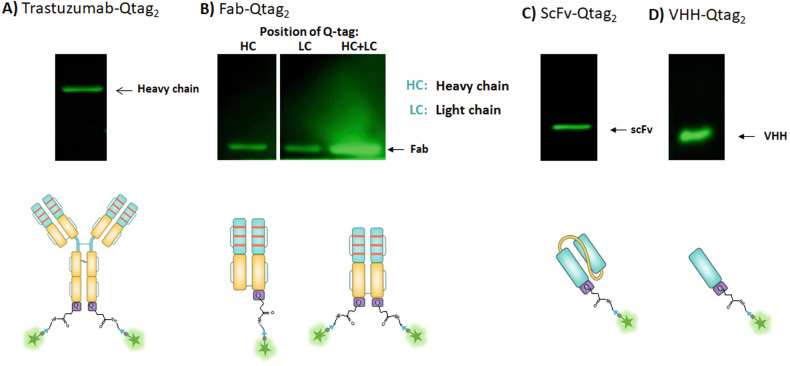
Microbial transglutaminase mediated site-specific conjugation of antibody drug conjugates. SDS-PAGE analysis and fluorescence detection of the incorporation of alexa488-cadaverine (amine donor) into Her2 trastuzumabQtag2 (**A**), Fab- Qtag2g (**B**), scfv- Qtag2 (**C**) and VHH- Qtag2 (**D**) using mTG.

To evaluate the relative efficacy of conjugation, Qtag_2_ was engineered in the C-terminal domains of the anti-Her2 antibody fragments (Fab, scfv and VHH) (Fig. 2B). The observed fluorescent signals at the HC and LC suggest that the investigated location does not affect the conjugation efficacy. In addition, the higher labeling level of the Fab with double Qtag indicates that these engineered Fabs are appropriate for the generation of DAR1 as well as DAR2. Using scfv (Fig. 2C) and VHH fragment (Fig. 2D), the same process was performed by addition of a sequence Q-Tag_2_ at the C-terminus domain.

### Immunoreactivity and internalization of the conjugates

To verify that the conjugation has no adverse effect on the antigen binding, Immunoflurescence assay, flow Cytometry and Biacore surface plasmon resonance (SPR) analysis was performed using rh-HER2 protein (Table 2). The Kd studies showed that the Trastuzumab Qtag_2_, lysine deleted or ADC do not show significant difference (92.1 pM, 66.2 pM, 85.3 pM, respectively) compared to the reference antibody [26].

**Table 2:** Functional analysis of antigen binding properties.

Antibody	Kd (pM)
*Trastuzumab-Qtag*_*2*_	92.1 ± 3.1
*Trastuzumab*_*Kdel*_*-Qtag*_*2*_	66.2 ± 2.9
*Trastuzumab*_*Kdel*_*-Qtag*_*2*_*-PEG*_*4*_*-VC-MMAE*	85.3 ± 2.8

Biacore surface plasmon resonance analysis of anti-human IgG FC captured (AHC biosensor) recombinant antibodies and conjugates with rhu-HER2 protein. KD, affinity constant.

To study whether the conjugation interferes with the antigen binding, the cadaverine derivate of AlexaFluor 488 was conjugated to full length antibody, Fab, scfv and VHH formats. The antigen binding ability of the conjugates was examined by immunofluorescent staining and flow cytometry. Cell lines with high (SKBR3) and normal (MCF7) expression level of HER2/neu receptor were included in the assay.

**Fig. 3:**
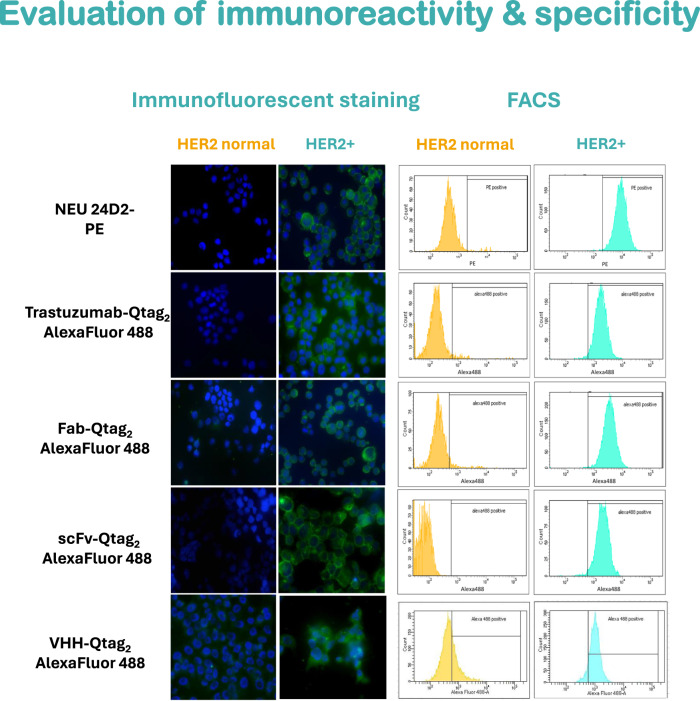
Immunofluorescent staining (left panel) and fluorescent activated cell sorting (FACS) analysis (right panel) on SKBR3 (HER2/neu positive) and MCF7 (HER2/neu status normal) cells. Trastuzumab-Qtag_2_, Fab-Qtag_2_, scFv-Qtag_2_ and VHH-Qtag_2_ were conjugated with AlexaFluor488-cadaverine and the antigen binding profile was compared to the commercially available Neu-antibody(24D2)-PE. Green, AlexaFluor488 or PE signal; blue, DAPI (nucleus).

Each antibody format showed very similar staining profile on HER2/neu positive cells, comparable to the commercially available control antibody (Neu242-PE). In addition, target selectivity was confirmed by negative staining of the cell that express normal level of HER2/neu receptor (Fig. 3, left panel). Immunoreactivity and specificity were further demonstrated by FACS analysis (Fig. 3, right panel).

As expected, these results prove that the site-specific conjugation preserve the functionality of various antibody formats, since the conjugation site is distant from the antigen binding site.

**Fig. 4:**
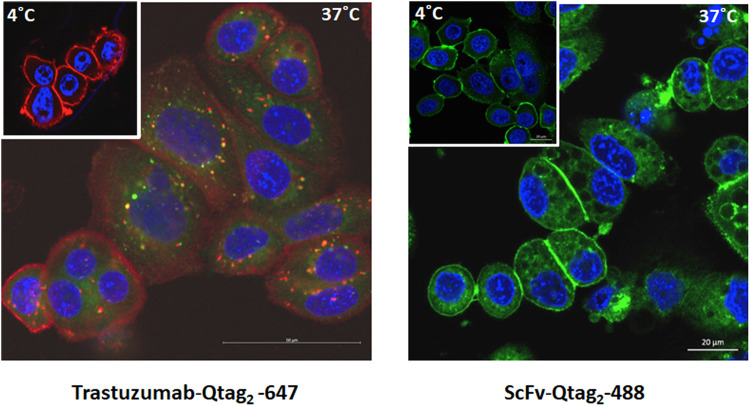
Immunofluorescent staining of breast cancer cell lines: SKBR3 (HER2/neu positive). Evaluation of intracellular internalization of AlexaFluor674 conjugated Trastuzumab-Qtag2 (left panel) or scfv-Qtag_2_ (right panel) at 4 °C or 37 °C using fluorescent microscopy. Red, AlexaFluor674 signal; green, lysotracker; blue, DAPI (nucleus). AlexaFluor 488 scfv (right panel) at 4 °C or 37 °C using fluorescent microscopy. Green, AlexaFluor 488 – scfv; blue, DAPI.

Furthermore, the antigen binding ability and internalization of AlexaFluor647-cadaverine conjugated Trastuzumab-Qtag to HER2/neu receptor was examined by immunofluorescent staining on HER2/neu positive (SKBR3). Specific recognition of HER2/neu receptor on the surface of HER2/neu expressing cells but not on target negative cells was performed by labeling at 4 °C (Fig. 4 left pannel). In addition, to examine the intracellular trafficking properties, the lysosomes were visualized by acidotropic green fluorescent probe (Lysotracker). Rapid internalization of AlexaFluor647-cadaverine conjugated Trastuzumab-Qtag_2_ (red color) was observed at 37 °C after 10 min, followed by the transported to endosomes and lysosomes, leading to yellow merging co-localization (Fig. 4 left pannel). This observation indicates that cellular processing of the antibody is not altered by the conjugation, thus, that the attached compound can be released.

A similar internalization assay was performed using AlexaFluor488-cadaverine conjugated Trastuzumab-Qtag_2_ scfv (green color). A clear membrane signal is observed at 4 °C corresponding to the scfv binding to the receptor at the cell membrane (Fig. 4 right pannel). At 37 °C after 10 min a strong signal is observed inside the cell confirming the scfv internalization (Fig. 4 right pannel).

Corresponding to our expectations these results prove that the site-specific conjugation preserves the functionality of the ADC including the antibody fragment, since the conjugation site is distant from the antigen-binding site.

### In vitro cytotoxicity

The functionality of ADCs was tested by the evaluation of their in vitro cytotoxicity and target selectivity on HER2/neu overexpressing (SKBR3, BT-474) and HER2/neu negative (MBA-MB-231) cell lines. Cell killing potency of our conjugates was compared to the commercially available T-DM1 (Kadcyla®). Cells were plated in 96 wells plate, a serial dilution of free drugs and ADC conjugated format was applied and incubated for 3 days. Toxicity was using Sulforhodamine B (SRB) colorimetric assay.

As presented in Table 3, potent dose dependent cytotoxicity of ADCs was obtained on HER2/neu expressing cell lines. No effect on target negative cell line was observed. The unconjugated DM1 and MMAE both killed HER2/neu positive and negative cells with an IC_50_ between ∼1–20 nM for SKBR3 (DM1: 1.15 ± 0.07 nM, MMAE: 1.93 ± 0.07 nM) and MBA-MB-231 (DM1: 2.78 ± 1.75 nM, MMAE: 20.69 ± 7.75 nM) cells, whereas they were approximately 100-fold less potent on BT-474 cells (DM1: 118 ± 1.5 nM, MMAE: 125 ± 1.2 nM). These results prove the specific toxicity of ADCS mediated through HER2/neu binding.

**Table 3:** In vitro killing of ADCs.

Cell lines/IC50 (nM)	T-DM1	Trastuzumab Qtag2 -DM1	VHH Qtag2-DM1	scfv-Qtag2-DM1	DM1
SKBR3 (HER2/neu+)	0.088 ± 0.01	0.363 ± 0.02	4.364 ± 0.07	1.21 ± 0.04	3.039 ± 0.07
BT-474 (HER2/neu+)	∼0.02 ± 2.1	>10	10.1 ± 0.33	>10	112.9 ± 5.8
MDA-MB-231 (HER2/neu-)	>10	>10	3.2 ± 0.56	>1000	2.78 ± 1.75

Cell lines/IC50 (nM)	Trastuzumab Qtag2 -MMAE	MMAE
SKBR3 (HER2/neu+)	0.163 ± 0.07	3.95 ± 0.66
BT-474 (HER2/neu+)	∼0.65 ± 10.21	147.9 ± 6.25
MDA-MB-231 (HER2/neu-)	>10	20.69 ± 7.75

A cytotoxicity assay was performed to evaluate inhibitory effect of free and conjugated drugs on cell proliferation. Two types of HER2/neu positive (SKBR3 and BT-474) and MDA-MB-231 a target negative cell line were used in the experiment. Three days after the addition of the drugs, cell death was quantified by sulforhodamine B assay. IC50 values were determined by fitting of non-linear regression curves to the data using GraphPad Prism 7.0b (GraphPad Software Inc., San Diego, CA, USA) software. Data points represent the mean of two independent experiments. T-DM1, Ado-Trastuzumab emtansine (Roche); Trastuzumab-Qtag2-PEG4-SMCC-PAB-DM1; Trastuzumab-Qtag2-PEG4-VC-PAB-MMAE.

*ND* non-determined.

On SKBR3 cells, in agreement with the published data [27], comparable IC_50_ values with the benchmark T-DM1 was obtained (T-DM1: 0.078 ± 0.01 nM, Trastuzumag-Qtag_2_-DM1: 0.32 ± 0.02 nM, Trastuzumag-Qtag_2_-MMAE: 0.16 ± 0.02 nM) (Table 3). These values indicate a positive proportional correlation between drug loading and cell cytotoxicity in vitro. Similarly, to the free drugs, lower antiproliferative activity of ADCs on BT-474 cells was detected (Table 3).

### In vivo biodistribution and tumor homing

**Fig. 5:**
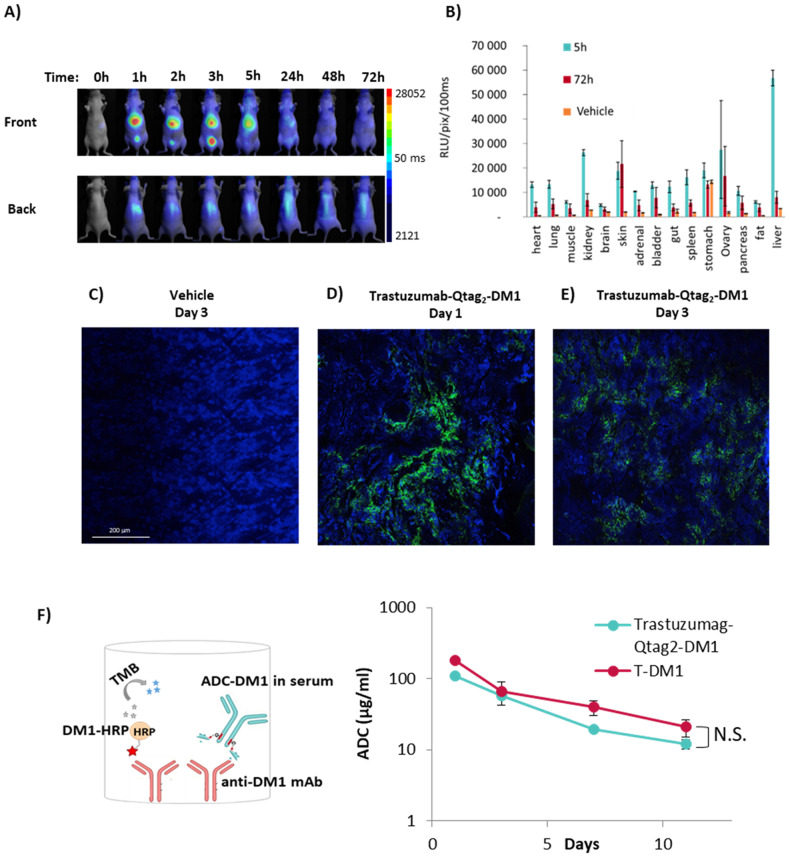
Biodistribution of ADC. **A** 2D in vivo fluorescent images of NMRI Nudes mice and **B** ex vivo fluorescent signal of organs after intravenous injection of AlexaFluor 680 labeled Trastuzumab-Qtag2-MMAE (Mean ± SEM (*n* = 4)). Immunofluorescent analysis of BT-474 tumor sections after intraperitoneal injection of **C** vehicle control or Trastuzumab-Qtag2-DM1 **D** on day 1 and **E** day 3. The green signal represents the homing of ADC using AlexaFluor488 labeled anti-DM1 mouse monoclonal antibody. Blue, DAPI (nucleus). **F** Quantitation of the ADCs concentrations in mouse serum (15 mg/kg of ADC, BT-474-Her2 positive tumor) was performed by competition ELISA assay. 96-well plates were coated with anti-DM1 mAb to capture ADC-DM1 and HRP-labeled DM1. The detected absorbance is inversely proportional to the amount of ADC-DM1 conjugate in the test sample (Mean ± SEM (*n* = 3). Statistical significance was evaluated using GraphPad Prism 7.0b (GraphPad Software Inc., San Diego, CA, USA) software using unpaired one tailed *t*-test, N.S. non significative.

To evaluate in vivo biodistribution of ADC in mice, Trastuzumag-Qtag_2_-MMAE was labeled with 680 nm dye (AlexaFluor680) by NHS ester reaction. The protocol was optimized to yield in approximately 1.2 AlexaFluor 680 per ADC in order to minimize the effect (affinity and photophysical properties) [23, 28] of the fluorophore conjugation. Figure 5A shows the 2D fluorescent images of mice at various time points after intravenously injection (0, 1, 2, 2, 5 24, 48, 78 h). Rapid systemic dispersion was observed in 1 h after injection. Then, it was eliminated by bladder, thus long-term retention of AlexaFluor680-ADC was not observed, indicating low toxic index. To confirm tissue distribution, mice were euthanized and the ex vivo fluorescent signal of the organs was analyzed at 5 and 72 h post injection. As shown in Fig. 5A, B, an important uptake was observed during the first 5 h post injection in liver, kidney and bladder, whereas other organs showed minimal accumulation (Fig. 5B). The persistent signal level in the skin might be correlated to the high micro vascularization of this organ. The tissue distribution pattern profile is similar to the published data [29].

Tumor homing of Trastuzumab-Qtag_2_-PEG_4_-SMCC-PAB-DM1 was confirmed by immunofluorescent staining. HER2/neu positive tumor bearing Balbc/mice were intraperitoneally injected with the ADC (15 mg/kg), and tumors were collected at day 1 and day 3 post injection. Confocal microscopy analysis of tumor cryosections with vehicle (Fig. 5C) or AlexaFluor488 labeled anti-DM1 mAb is shown in Fig. 5D. Strong specific staining of Trastuzumab-Qtag_2_-PEG_4_-SMCC-PAB-DM1 could be observed after 1 day, revealing the efficient homing of the ADC to the tumor. Whereas 3 days post injection the intensity of the fluorescent signal was weaker and the staining pattern was more distributed (Fig. 5E). This result can indicate the perfusion of the ADC and the released cytotoxic compound in tumor site. The pharmacokinetic of T-DM1 and Trastuzumab-Qtag_2_-DM1 was followed during 12 days the remaining antibody was titrated in the mice sera based on quantitative ELISA assay (Fig. 5F). No significant difference between TDM-1 and the Trastuzumab-Qtag_2_-DM1 was observed.

### In vivo anti-tumor activity

**Fig. 6:**
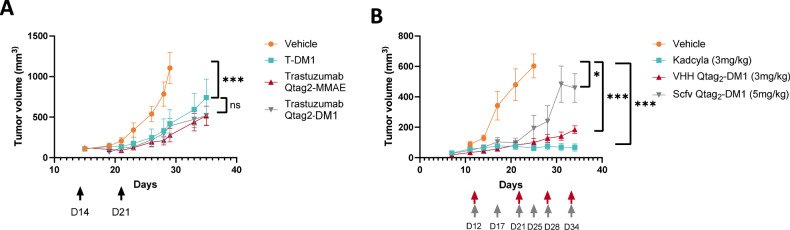
In vivo evaluation of ADCs in a mouse ectopic xenograft model. The in vivo efficacy was investigated in SCID mice implanted subcutaneous with BT-474 HER2-positive mammary tumors. **A** Tumor response was evaluated using single administration of 3 mg/kg dose (trastuzumab ADC or T-DM1) and tumor volume was measured at different time intervals. **B** Tumor response was evaluated using several administrations of various dose of antibody fragments (trastuzumab -Qtag2 -DM1 or T-DM1) and tumor volume was measured at different time intervals Mean ± SEM (*n* = 6). Tumor size was followed over 3 weeks. Statistical significance was calculated by unpaired one tailed *t*-test using GraphPad Prism 7.0b (GraphPad Software Inc., San Diego, CA, USA) software;. n.s : non significative, *P < 0.05 ***P < 0.001.

The therapeutic activity of Trastuzumab-Qtag_2_-DM1 and Trastuzumab-Qtag_2_-MMAE was followed in HER2/neu overexpressing breast cancer mouse xenograft model and compared to commercially available T-DM1. SCID mices were implanted subcutaneous with BT-474 cell derived mammary tumors. Fourteen days after, the animals were randomized into groups with a mean tumor volume of 100 mm^3^ and submitted twice (day 14 and day 21) to IP administration of 3 mg/kg dose each ADC. Tumor volumes were measured at different time intervals during three weeks. Compared to the vehicle control, the treatment led to inhibition of tumor growth for several days, then the tumors continued to grow (Fig. 6A).

In order to evaluate the efficacy of smaller formats, SCID mice were implanted subcutaneous with BT-474 cell derived mammary tumors. Twelve to 14 days later, when the tumors reach 50–100 mm^3^, mice were randomized into groups and submitted to different protocols: either IP injection of scfv-Qtag_2_-DM1 formats or T-DM1 at 3 mg/kg once a week for 4 weeks or IP injection of VHH-Qtag_2_-DM1 twice a week for 4 weeks (Fig. 6B)

These results highlighted for the first time, the ability of VHH formats to inhibit tumors growth as the clinically used T-DM1.

## Discussion

The CovIsolink technology was validated by various analytical methods. A DAR 2 was obtained using small hydrophilic molecule Alexa 488 and a DAR 1.8 was obtained for the hydrophobic molecule MMAE on full length antibody without DAR 0.

No interference or loss of immunoreactivity was found for all mabs and antibody fragments. Interestingly, for in vitro validation, Trastuzumag-Qtag_2_-MMAE exhibited higher toxicity than DM1 conjugates. Since there was no significant difference between DM1 and MMAE as an unconjugated format, we can hypothesize that the effect is due to the linker features, which act through the intracellular trafficking mechanism of HER2/neu receptor. Once more, Trastuzumab-Qtag_2_-MMAE contains the protease-sensitive cleavable valine-citrulline (VC) dipeptide linker that is released by cathepsin B in the endosomes or lysosomes. In case of Trastuzumag-Qtag_2_-DM1 and T-DM1 the complete lysosomal degradation of the antibody is required after internalization due to the non-cleavable SMCC linker. Thus, efficacy of the cytotoxic compounds can be altered by the recycling properties of the receptor. In this recent study these properties were not further investigated.

The in vivo studies demonstrated that even if T-DM1 has more drug molecules conjugated to the antibody (approximately threefold), both of ADCs exhibited very similar in vivo efficacy. This observation correlates with the previous studies, demonstrating that unlike in vitro cell cytotoxicity the higher DAR does not improve therapeutic activity [30]. Trastuzumab-Qtag_2_-MMAE stabilized the tumor growth (cytotoxic effect) over a longer period as DM1 conjugates. Moreover, we observed lower heterogeneity in the Trastuzumab-Qtag_2_-MMAE conjugate treated groups. In addition, no significant difference (*p* value 0.068) between the wt and Kdel variant conjugated formats could be detected (data not shown). These results show that our site-specific full formats conjugates demonstrated comparable in vivo potency to the clinically used T-DM1.Interestingly, even if T-DM1 has much more drugs per molecule (sixfold), VHH-Qtag_2_-DM1 formats, with only one drug per molecule, harbor similar anti-tumor efficacy compared to T-DM1. On the other hand, even if scfv-Qtag_2_-DM1 formats allow to delay the tumors growth, all tumors relapses. The relative low efficacy of scfv-Qtag_2_-DM1 may be explain by it’s low solubility and rapid clearance. Indeed this format is less stable and had a tendency to form aggregates because of the lack of FC domain and the small size decrease their half-life under 1 day versus 3 weeks for full length antibodies [31]. Even if CovIsoLink technology succeed to cross links DM-1 successfully with a high efficacy, the physico-chemical properties of scfv confirms the low efficacy as scfv ADC. To the best of our knowledge, scfv are not used alone as ADC for clinical trials. However, the scfv format remain a powerful tool for cancer diagnosis or therapy such as CAR-T cells therapy [32], using immunoliposomes [33], or to be used in bi-specific antibodies [34]. This fragment seems to be more convenient for diagnosis and targeting instead of drug vectorization. Contrary to the scfv format, VHH have better solubility and are easier to conjugate at higher concentration without precipitation. Nevertheless, their very small size required to be injected more frequently to be efficient [35] due to the renal clearance but there efficacy is better compared to scfv.

To conclude, using a novel glutamine peptide substrate, we developed CovIsoLink^TM^ technology for the mTG-mediated site-specific conjugation of antibodies. As a proof of concept the peptides were engineered into the sequence of Trastuzumab targeting HER2/neu receptor. We confirmed that the immunoreactivity and internalization is not alerted by the conjugation. Moreover, no unconjugated species could be observed, confirming the optimized potential of this technology for larger GMP batch production requiring less purification processes. Furthermore, compare to T-DM1, which is already used in clinic, similar in vitro and in vivo tumor cell killing potency was demonstrated, even with lower average DAR, suggesting lower risk of toxicity with similar efficacy. Thus, our technology provides the rationale for a suitable alternative enzymatic conjugation strategy of different payloads on optimized peptide to produce homogeneous batches, without unconjugated species including antibody fragment such as scfv or VHH.

## Materials and methods

### Construction of recombinant antibodies with glutamine tag

The variable region sequence of Trastuzumab (clone 4D5) was cloned into TGEX-LC (human kappa) and TGEX-HC (human IgG1) mammalian expression vectors (Antibody Design Laboratories, USA) by restriction cloning (LC: BspEI and BsaI, HC: BssHII and BsmBI, Thermo Fisher Scientific, France). The amino acid sequences of different glutamine-tag substrates (LLQG, Tag2, Tag3, Tag4, Tag5) specific for microbial transglutaminase were introduced into the C-terminal part of the heavy chain by overlap extension invers polymerase chain reaction (PCR). The sequences of the peptide tags are not shown, since they are currently patented. Additionally, the heavy chain C-terminal lysine453 was deleted by site directed mutagenesis.

The primers were designed with OligoAnalyzer 3.1 (www.idtdna.com) and synthesized by Eurogentec (polyacrylamide gel electrophoresis (PAGE) purification). Fifty microliters PCR reaction was composed of 0.5 µl template (5 ng), 2.5 µl of each primer (10 µM), 1 µl dNTP mixture (10 mM) (Promega, France), 10 µl 5× Phusion HC buffer (New England Biolabs, France), 1.5 µl DMSO (New England Biolabs, France), 31.5 µl nuclease free H_2_O (B. Braun, France) and 0.5 µl Phusion high-fidelity DNA polymerase (2000 U/ml) (New England Biolabs, France). The PCR cycling parameters were 98 °C 30 s, (98 °C 10 s, 72 °C 1.5 min) × 30 cycles and 72 °C 7 min.

Following the PCR reaction, 2 µl DpnI (10 U/µl) (Thermo Fisher Scientific, France) was added and the mixture was incubated at 37 °C for 4 h to degrade the original unmodified plasmid templates. After DpnI digestion, 5 µl of the mixture was used to transfect low recombination NEB 5-alpha competent *Escherichia coli* cells (New England Biolabs, France) according to the manufacturer’s instructions. Plasmid DNA was isolated using PureYield Miniprep Kit (Promega, France) and sequenced.

### Expression and purification of recombinant antibodies

Recombinant full length and Fab antibodies (rAbs) were transiently expressed in HEK293AD cells (ATCC). Cotransfection of LC and HC plasmids were carried out with Lipofectamin 3000 (Thermo Fisher Scientific, France) in Opti-MEM reduced serum media (Thermo Fisher Scientific, France) according to the manufacturer’s instructions.

scfv and single domain antibodies were transformed in BL 21 bacteria

Cell culture supernatants were purified by protein A-sepharose (GE Healthcare, France), washed with 1× PBS (140 mM NaCl, 2.7 mM KCl, 10 mM Na_2_HPO_4_, 1.8 mM KH_2_PO4), eluted with 0.1 M glycine pH3 and immediately neutralized with 1 M Tris-HCl pH 8. The buffer was exchanged against 1× PBS with Zebaspin desalting column (MWCO 40 kDa, Thermo Fisher Scientific, France). The purity of the rAbs was assessed by Sodium Dodecyl Sulfate polyacrylamide gel electrophoresis (SDS-PAGE) and silver-nitrate staining. The average yield of the purified rAbs was 10 mg/L.

Antibody-binding fragment (Fab) fragment with C-terminal heavy chain glutamine tag was created from TGEX-VH_4D5_-HC-Qtag_2_ (human IgG1) vector, encoding the variable region of Trastuzumab (clone 4D5) and the sequence of Q tag, by the deletion of CH2 and CH3 domains. For the construction of C-terminal light chain glutamine tagged Fab, overlap extension invers PCR was carried out with TGEX-VL_4D5_-LC (human kappa) vector. The cloning vector of single chain variable fragment (pET23NN-scfv_4D5_-LLQG tag) was kindly supplied by Pierre Martineau (Institut de Recherche en Cancérologie de Montpellier, France). LLQG tag was substituted to Q tag by overlap extension invers PCR.

The Single domain antibody (Clone A10 Q tag) was a kindly gift from Franck Perrez, Curie institute, Paris

### Enzymatic conjugation

For conjugation of antimitotic agents, amine-PEG4-VC-PAB-MMAE and amine-PEG4-SMCC-DM1 (reconstituted in DMSO; Sigma, France) were pre-incubated with 1 mM triethylamine (Sigma, France) for 10 min at room temperature (RT) with gentle shaking. The final antibody concentration was adjusted to 10 mg/ml and incubated with 30-fold (MMAE) or 10-fold (DM1) molar excess of the drugs in the presence of microbial transglutaminase (17 U/mg antibody; Zedira, Darmstadt, Germany) in PBS buffer. The reaction was performed overnight (O/N, 16 h) at room temperature (RT) with gentle agitation. The excess of the payloads and the enzyme were removed by protein A purification then the buffer was exchanged buffer against 1× PBS with Zebaspin desalting column (MWCO 40 kDa, Thermo Fisher Scientific, France). The products were analyzed by LC/MS.

The conjugation of fluorophores was carried out in PBS buffer, containing 0.25 mg/ml of antibody,30-fold molar excess of AlexaFluor448-cadaverine or AlexaFluor647-cadaverine (Thermo Fisher Scientific, France) and 4 U/mg antibody of microbial transglutaminase. Following O/N incubation with gentle shaking at RT, the tagged antibodies were purified by protein A (detailed in section Expression and purification of recombinant antibodies). Fluorescent labeled conjugates were analyzed on 10% SDS-PAGE and visualized by UV-fluorescent detection and Silver-nitrate staining.

### MS analysis (DAR Calculation)

Mass spectrometric analyses of ADCs were performed on a Bruker maXis mass spectrometer coupled to a Dionex Ultimate 3000 RSLC system. Prior to MS analysis, samples (ca. 5 µg) were desalted on a MassPREP desalting cartridge (2.1 × 10 mm, Waters) heated at 80 °C using 0.1% formic acid as solvent A and 0.1% formic acid in acetonitrile as solvent B at 500 µL/min. After 1 min, a linear gradient from 5 to 90% B in 1.5 min was applied; the first 1.5 min were diverted to waste. HRMS data were acquired in positive mode with ESI source over the m/z range from 900 up to 5000 at 1 Hz and processed using DataAnalysis 4.4 software (Bruker) and MaxEnt algorithm for spectral deconvolution.

### Immunoreactivity analysis (Surface Plasmon Resonance)

Binding kinetics and affinity of wild type (Ab-Qtag) and heavy chain C-terminal lysine deleted rAb with engineered Tag2 (K453delAb-Qtag) or MMAE conjugated format (K453del-ADC-MMAE) to recombinant human Her2 protein (rh-Her2, Acro Biosystems, USA) was measured by Surface Plasmon Resonance method on Octect system (PALL FortéBio, France). R Abs were diluted in 1x kinetics buffer (PALL ForteBio, France) to achieve a final concentration of 5 µg/ml and were immobilized onto anti-uman IgG Fc capture (AHC) biosensor (PALL ForteBio, France). Rh-Her2 was injected for 600 s ranging from 2 nM to 20 nM (diluted in 1× kinetics buffer) and association/dissociation was monitored for 300 s. Double-referenced binding curves were subtracted to prevent system artifacts and non-specific binding, then binding curves were fit to 1:1. The affinity constants (*K*_D_) were calculated from the ratio of the association and dissociation rate constants (*k*_on_/*k*_dis_).

### Internalization assay

MDA-MB-231 cells and SKBR3 cells are plated in a 96 wells plate at 7500 cells per well. After 36 h, cells were washed with 100 µl of Fluorobright DMEM (Life technologies, France) to remove the phenol red. The lyso-tracker dye (Life technologies, France) was diluted 10,000 times, the cell mask dye was diluted 1000 times and were incubated together 30 min at 37 °C. The nucleus staining was performed with 0.5 µg/ml of Hoechst 3342 (Life technologies, France) and then incubated 10 min at 37 °C. The plate was washed again with 100 µl of fluorobrite DMEM. The antibody was injected and the kinetic was immediately recorded at 37 °C by an HCS Operetta microscope (Perkin Elmer) during 3 h. Control were performed on ice in order to block the cell metabolism. After 3 h the plate was washed with PBS and the cells were fixed with 150 µL of paraformaldehyde at 4 °C for 15 min. The plate was washed with 150 µl of PBS and imaged by confocal microscope (LSM 780 Confocal microscope, Zeiss, France).

### In vitro cytotoxicity assay

In vitro cytotoxicity of CovADC-DM1 and CovADC-MMAE was compared to T-DM1 (Kadcyla, Roche, France) on BT-474 and SKBR3 Her2-positive breast cancer cell lines, MDA-MB-231 Her2-negative cell line was included in the assay to confirm target selectivity. All cell lines were obtained from ATCC and cultured in DMEM supplemented with 10% FBS, 100 U/ml penicillin/streptomycin and 2 mM l-glutamine (Life technologies, France). Cells were maintained at 37 ˚C with 5% CO_2_ atmosphere. Optimal cell density of each cell lines was determined by IncuCyte S3 live-cell analysis system (Essence BioScience, UK). Cells were plated (SKBR3 and BT-474: 5000 cells per well, MDA-MB-231: 1,250 cells per well) on 96-well plates. Following the incubation for 24 h, cell culture medium was removed and serial dilutions of ADCs and free unconjugated drugs were added in triplicates. After 3 days incubation cytotoxicity was evaluated by sulforhodamine B assay (SRB assay kit, Sigma, France) based on the measurement of cellular protein content according to the manufacturer’s instructions. Briefly, cells were fixed with 50% (wt/vol) TriChloroacetic Acid (TCA) at 4 °C (30 µl/well, final concentration 11.5%) for 1 h. After washing with dH_2_O cells were stained with 100 µl of 0.04% (wt/vol) SRB solution for 30 min and subsequently washed with 1% (vol/vol) acetic acid to remove unbound stain. Cellular protein bound dye was solubilized in 200 μl of 10 mM Tris base solution (pH 10.5) and the absorption was measured at 510 nm (Microplate reader, Tecan, Switzerland). The raw data was processed using GraphPad Prism 7.0 (GraphPad Software, San Diego, USA). Error bars in graphs represent SEM of two measurements.

### Biodistribution

Anti-Her2 CovADC-MMAE (2 mg/ml) was conjugated with DMSO dissolved Alexa Fluor 680-NHS ester (10 mg/ml) (Thermo Fisher Scientific, France) at a molar ratio of 1:44 in 0.1 M bicarbonate buffer pH 9 for 1 h at RT. Immunoconjugate was purified by protein A-sepharose (GE Healthcare, France) and the buffer was exchanged against 1× PBS. The degree of labeling was determined by spectrophotometry.

6 weeks-old NMRI Nude female mice (*n* = 11) were intravenously injected with 100 µg of CovADC-MMAE-alexa680 conjugate or vehicle control (PBS). In vivo 2D fluorescent images were acquired at the different time points (0, 1, 2, 3, 5 h (*n* = 6) and 24, 48, 72 h (*n* = 3) post injection) with Fluobeam700 (Fluotipcs, Optimal, Grenoble, France). Mice were sacrificed after 5 h (*n* = 3) and 72 h (*n* = 3) post injection, tissues of interest (heart, lungs, muscles, kidneys, brain, skin, adrenal, bladder, gut, spleen, stomach, ovary, pancreas, fat, liver) were removed and ex vivo 2D fluorescence was detected followed by the quantification of mean pixel values.

### Tumor homing study

5 × 10^6^ of human breast carcinoma derived BT-474 cells were implanted subcutaneously into 6-week-old female SCID-CB17 mice (Charles River Laboratories, France). When the tumor reached an average volume of 100 mm^3^, mice were intraperitoneally injected with vehicle control (PBS) or 15 mg/kg of CovADC-DM1 (2 mice per group). Animals were sacrificed on day 1 and day 3 after administration of ADC and tumors were collected. Following the preparation of tumor sections, immunofluorescent staining was performed. Frozen samples were fixed in 70% (v/v) ice cold ethanol for 30 min at −20 C min and then rehydrated in PBS for 30 min at RT. Staining with 0.5 µg of AlexaFluor488 labeled anti-DM1 mouse monoclonal antibody (mab0160, Covalab, France) diluted in PBS was carried out at RT for 1 h in a humid chamber and the slides were subsequently washed with PBS. Nuclei were visualized with DAPI (Vectashield Mounting medium with DAPI, Vector laboratories, CA, USA) diluted in fluorescence mounting medium (Vectashield Mounting medium, Vector laboratories, CA, USA). Tumor sections were imaged by confocal microscopy (LSM 780 Confocal microscope, Zeiss, France).

ADCs level in plasma were monitored at day 0, 3, 7 and 11 and quantified using ADC-DM1 kit (Kit005, Covalab, France).

### In vivo anti-tumor activity assay

In vivo efficacy studies of ADCs were performed in Her2/Neu expressing ectopic xenograft mouse model. SCID-B17 mice (Charles River Laboratories, France) were subcutaneously injected with 5 × 10^6^ of human breast carcinoma derived BT-474 cells,. Approximately 15 days after the tumor implantation, animals were randomized into groups with a mean tumor volume of 100 mm^3^ (*n* = 6 each). Tumor response was evaluated using intraperitoneal (IP) injection of either ADC formats or PBS as vehicle control according to the following protocols: 2 IP injection of 3 mg/kg full ADCs formats (cov-ADC-MMAE, cov-ADC-DM1, T-DM1), or 4 IP injection with 5 mg/kg for scfv formats (D12, D21, D28, D34), or IP injection every 3–4 days for VHH formats at 3 mg/kg (D12, D17, D21, D25, D28, D32). Tumor volume was measured in two dimension three times a week by caliper and calculated with the following formula: tumor volume = (4* *π* * *r*^3^)/3, where r median = average (length *A*/2; length *B*/2).

### Ethics statement

Animal experiments have been approved by the Ethics Committee for Animal Experimentation of the regional board CLB-ENS Lyon, France (Permit number: APAFiS# 11724 and DR2015-60).

### Statistical analysis

All data are presented as the mean value ± SD for in vitro studies and ± SEM for in vivo studies. Statistical analysis was performed using the unpaired one tailed *t*-test.

### Supplementary information

Supplementary material

Reproducibility Checklist

Original Data File

## Data Availability

Uncropped SDS-PAGE are available online as original data.
