# Supplementary material for: Antibody and antibody fragments site-specific conjugation using new Q-tag substrate of bacterial transglutaminase
Source: Cell Death Discov. 2024 Feb 15;10:79. doi: 10.1038/s41420-024-01845-3 (PMC10869684; doi:10.1038/s41420-024-01845-3)
Supplement: Supplementary file 1 — Supplementary material [file 41420_2024_1845_MOESM1_ESM.docx]

Supplementary Data

Figure S1 : DAR analysis by mass spectrometry:

Trastuzumab-Qtag2 Alexa488-cadaverine DAR 2 :

Trastuzumab-Qtag2 MMAE PEG4-VC-PAB DAR 1.5 :

Trastuzumab K453del-Qtag2 MMAE PEG4-VC-PAB DAR 1.74 :

Trastuzumab-Qtag2 DM1 PEG4-SMCC DAR 1.25 :
