# Supplementary figures and images for: Antibody and antibody fragments site-specific conjugation using new Q-tag substrate of bacterial transglutaminase
Source: Cell Death Discov. 2024 Feb 15;10:79. doi: 10.1038/s41420-024-01845-3 (PMC10869684; doi:10.1038/s41420-024-01845-3)

Figure 1

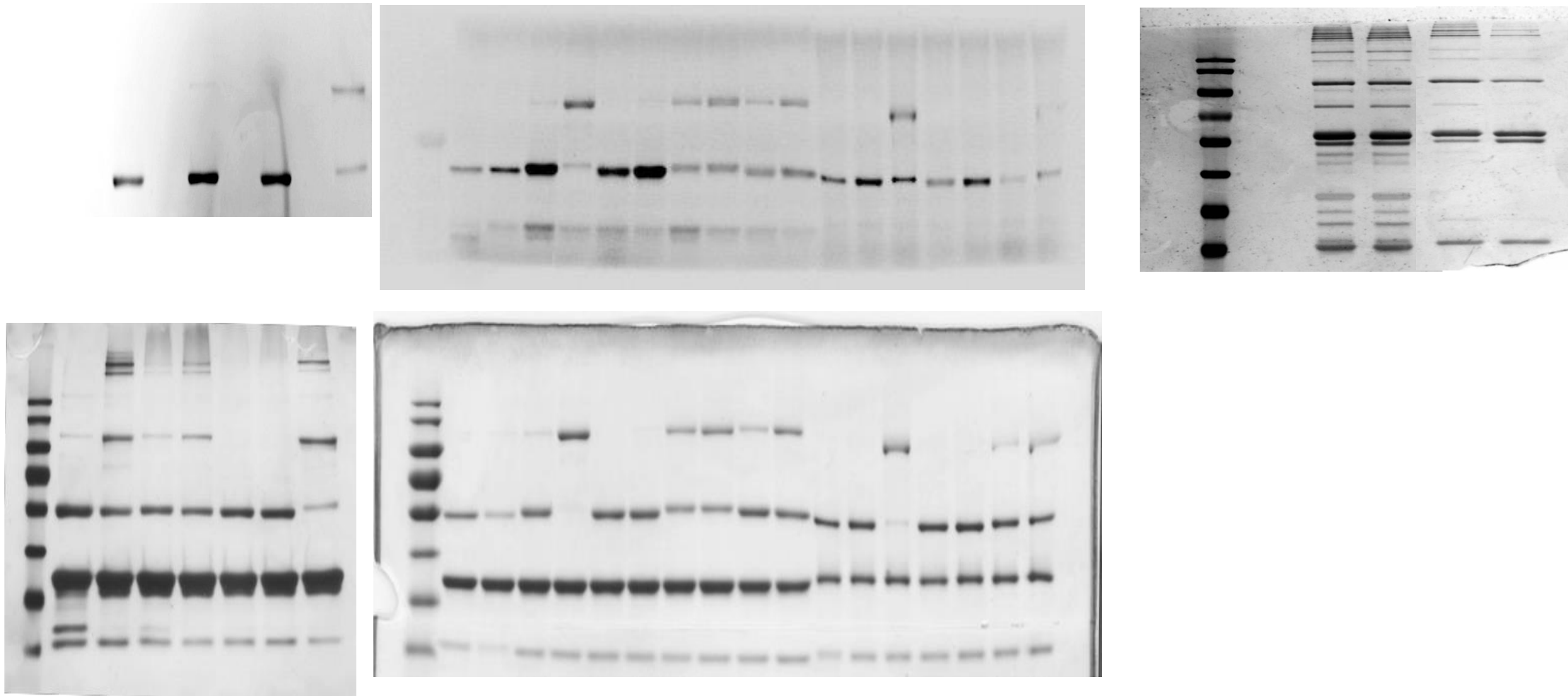

Figure 2 A)

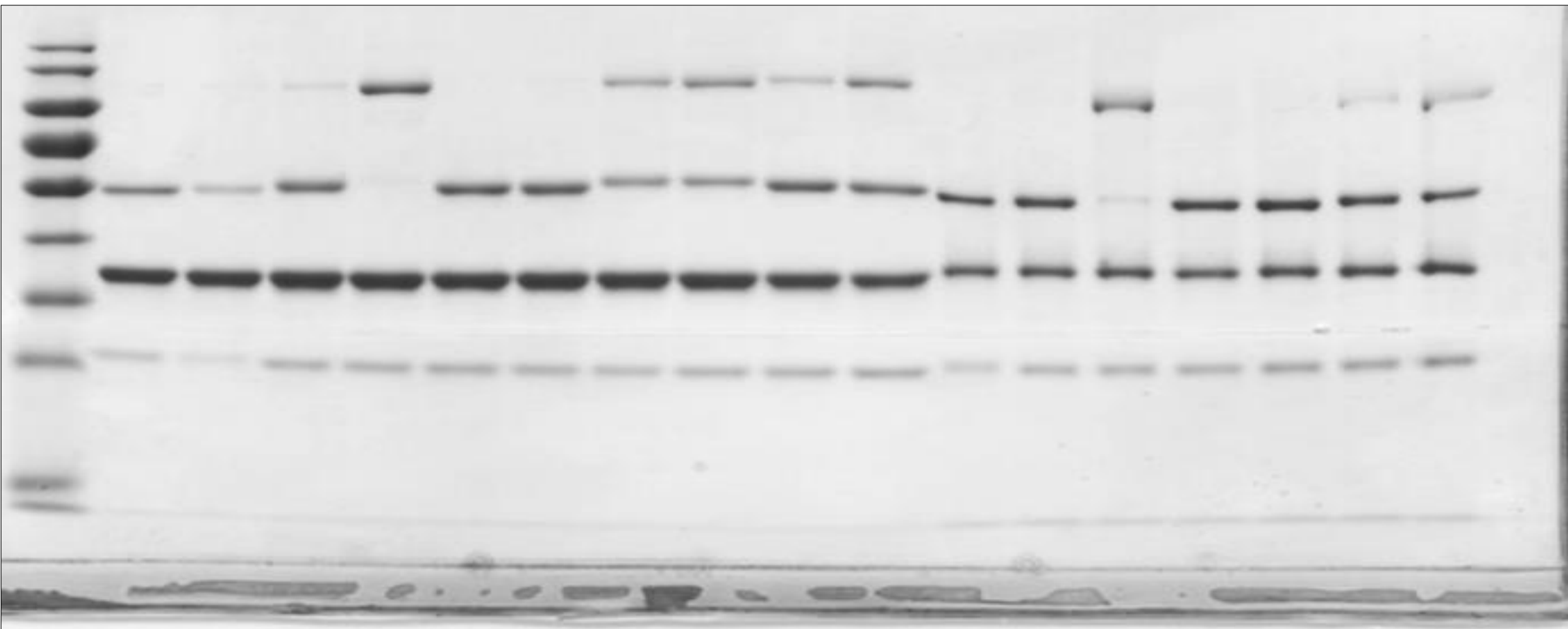

Figure 2 B)

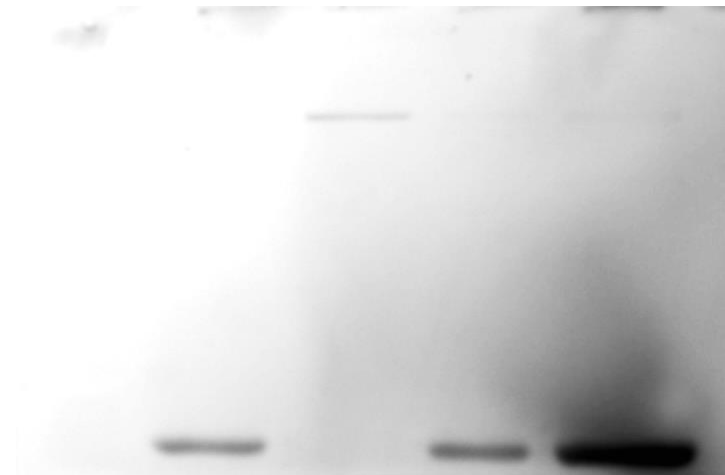

Figure 2 C)

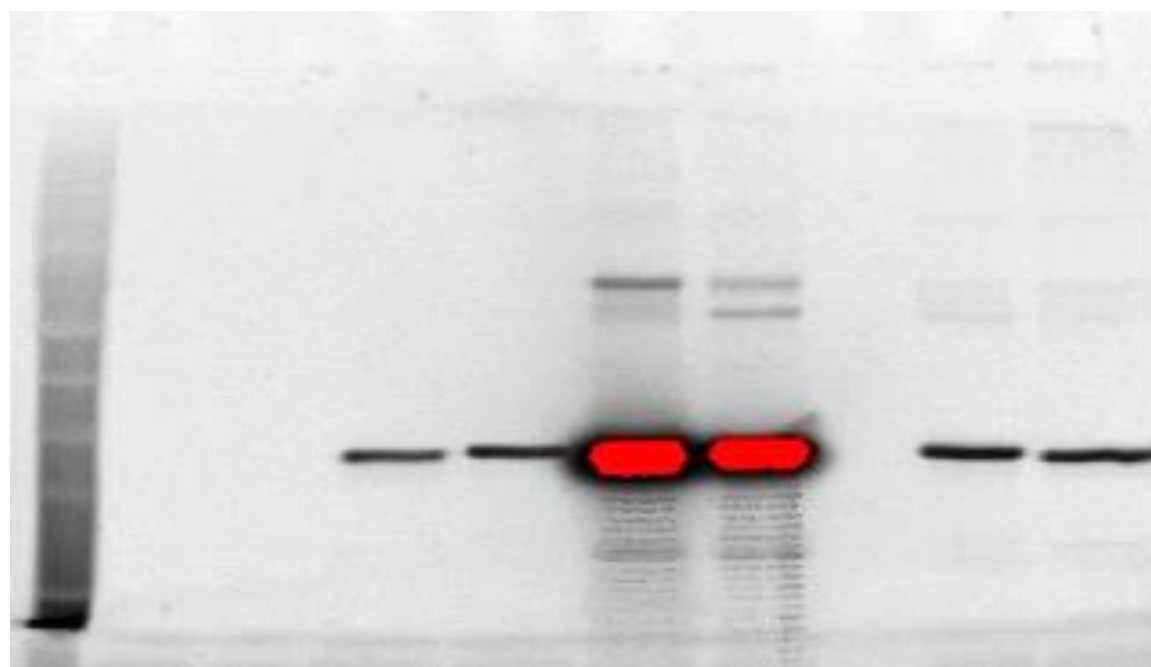

Figure 2 D)

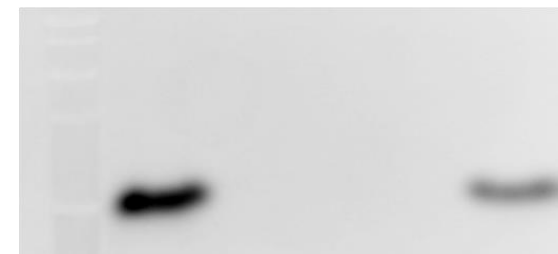

Supplement: Supplementary file 3 — Original Data File [file 41420_2024_1845_MOESM3_ESM.pdf]
